# Differentiation Potential of O Bombay Human-Induced Pluripotent Stem Cells and Human Embryonic Stem Cells into Fetal Erythroid-Like Cells

**DOI:** 10.22074/cellj.2015.489

**Published:** 2015-01-13

**Authors:** Fatemeh Ganji, Saeid Abroun, Hossein Baharvand, Nasser Aghdami, Marzieh Ebrahimi

**Affiliations:** 1 Department of Stem Cells and Developmental Biology at Cell Science Research Center, Royan Institute for Stem Cell Biology and Technology, ACECR, Tehran, Iran; 2 Department of Hematology, School of Medical Sciences, Tarbiat Modares University, Tehran, Iran; 3 Department of Regenerative Biomedicine at Cell Science Research Center, Royan Institute for Stem Cell Biology and Technology, ACECR, Tehran, Iran

**Keywords:** Induced Pluripotent Stem Cells, Differentiation, Hemangioblasts, Erythroid Cells

## Abstract

**Objective:**

There is constant difficulty in obtaining adequate supplies of blood components, as well as disappointing performance of "universal" red blood cells. Advances in somatic cell reprogramming of human-induced pluripotent stem cells (hiPSCs) have provided a valuable alternative source to differentiate into any desired cell type as a therapeutic promise to cure many human disease.

**Materials and Methods:**

In this experimental study, we examined the erythroid differentiation potential of normal Bombay hiPSCs (B-hiPSCs) and compared results to human embryonic stem cell (hESC) lines. Because of lacking ABO blood group expression in B-hiPSCs, it has been highlighted as a valuable source to produce any cell type *in vitro*.

**Results:**

Similar to hESC lines, hemangioblasts derived from B-hiPSCs expressed approximately 9% KDR^+^CD31^+^ and approximately 5% CD31^+^CD34^+^. In semisolid media, iPSC and hESC-derived hemangioblast formed mixed type of hematopoietic colony. In mixed colonies, erythroid progenitors were capable to express CD71^+^GPA^+^HbF^+^ and accompanied by endothelial cells differentiation.

**Conclusion:**

Finally, iPS and ES cells have been directly induced to erythropoiesis without hemangioblast formation that produced CD71^+^HbF^+^ erythroid cells. Although we observed some variations in the efficiency of hematopoietic differentiation between iPSC and ES cells, the pattern of differentiation was similar among all three tested lines.

## Introduction

Blood transfusions are universally used to treat various types of hematological diseases, such as hemoglobin abnormalities (sickle cell disease, thalassemia and methemoglobinemia) and abnormalities in the red blood cell (RBC) membrane or metabolism, as well astraumatic injury, surgery, treatment for burn victims and organ transplant recipients. To date, all blood components utilized for transfusion therapy are obtained through voluntary donation, which creates periodic shortages and concerns about the possibility of viral transmission of diseases via contaminated blood and blood products. Transfusions are also associated with other complications, many of which are immunological such as acute hemolytic reactions that occur with transfusion of RBCs. Moreover, ABO incompatible transfusion due to clinical or laboratory error remains the most widespread cause of transfusion related morbidity and mortality. Several studies have demonstrated that it is possible to enzymatically cleave A and B antigens to produce 'universal' RBCs, but to date this has not found widespread clinical application (1).

Thus, there is an emphasis on the need for better treatment methods, including hematopoietic cell replacement strategies as an alternative source for blood cells. Studies pioneered by Douay and Andreu (2) have demonstrated the feasibility of *in vitro* production of RBCs from CD34^+^ hematopoietic stem cells and progenitors that have been isolated from cord blood, bone marrow or peripheral blood. However, bone marrow or peripheral derived hematopoietic stem cells are difficult to expand and the possibility of using these cells for high scale industrial production of major blood components remains unresolved.

Pluripotent stem cells such as embryonic stem cells (ESC) and induced pluripotent stem cells (iPSCs) have been introduced as the best candidates to substitute for blood production *in vitro*. Human ESC (hESC) possess indefinite proliferative capacity in vitro, and have been shown to differentiate into all three germ layers that give rise to all type of somatic cells, including blood cells (3). In a comparison between ESCs and iPSCs, ethical issues do not avoid of iPSCs because they do not need embryonic or fetal material (4, 5) and they are more compatible because of autologous terminally differentiated somatic cells in mice and humans. Also iPSCs exhibit high similarity to ESCs due to effective proliferation and efficient differentiation into several cell types (6-8).

Previously, human iPSCs (hiPSCs) from donor fibroblasts derived from a Bombay phenotype have been established in our institute by ectopic expression of transcription factors that played a fundamental role in hESCs (9). The established cell line has been named Bombay hiPSCs (B-hiPSCs). According to mutational analysis, the Bombay phenotype fails to express the FUT1 and FUT2 genes by sequence analyses of fibroblasts and iPSCs which lead to lack of ABH antigen expression on blood cells, related to the ABO blood group system. The discovery of the Bombay phenotype, as a rare blood group, is an important discovery for the field of immunohematology. B-hiPSC-derived RBCs can be introduced as a histocompatible erythroid crucial for future cell therapy applications.

We sought to determine if differentiation of iPSCs into erythroid cells would follow the same patterns as that observed for hES cells. To achieve this goal we have used B-hiPSCs and two hES cell lines of various genomic sources, Royan H5 and 6 (RH5, RH6) and induced their differentiation into erythrocytes (10). The results revealed that cells produced in all lines were similar in the expression pattern of hemangioblast and erythroid progenitors regardless of their genomic diversity. Importantly, we observed in this system that hESCs differentiation closely resembled early human erythropoiesis development. In other word, sequential differentiation has been identified by formation of hemangioblast colonies. Afterwards, these colonies differentiated to erythroid cells that expressed hemoglobin F (α2γ2), however, they could not produce adult hemoglobin or hemoglobin A (α2β2).

## Materials and Methods

### Cell lines

In this experimental study, RH6 (44+XY), RH5 (44+XX) and BhiPSCs-11 (44+XY) with normal karyotype were used and cell passage number was between 30 and 40. B-hiPSCs-11 have been shown to be deficient in FUT1 and FUT2 genes expression was established at Royan Institute and maintained as undifferentiated cells in a feeder-free culture established previously by Larijani et al. (11).

### Adherent feeder-free and suspension culture of hiPS and hES cells

hiPS and ES cells were cultured on Matrigel (Sigma- Aldrich, E1270, USA) in serum-free media that consisted of Dulbecco’s modified Eagle’s medium (DMEM/F12, Gibco, 21331-020, USA) supplemented with 20% knock-out serum replacement (KOSR, Gibco, 10828-028, USA), 100 ng/ml basic fibroblast growth factor (bFGF, Royan Institute, Iran), 2 mM Lglutamine (Gibco, 25030-024, USA), 0.1 mM betamercaptoethanol (Sigma-Aldrich, M7522, USA), 1% nonessential amino acids (Gibco, 11140-035, USA), 100 IU/ml penicillin, and 100 mg/ml streptomycin (Invitrogen, USA). The suspension condition was used to expand undifferentiated iPS and hES cells, as follows. Briefly, cells were washed by Ca^2+^ and Mg^2+^- free phosphate-buffered saline (PBS) (Gibco, 21600- 051, USA), treated with ethylene glycol tetra-acetic acid (0.5 mM) (Sigma-Aldrich, E4378, USA) for 30- 40 seconds, incubated with 0.05% trypsin and 0.53 mM ethylenediaminetetraacetic acid (EDTA) (Gibco, 25300-054, USA) at 37˚C for 4-5 minutes, and then pipetted for 5-12 times. Then cells were transferred into low-attachment six-well plates (Corning-NY14831, USA) and treated with 10 μM ROCK inhibitor (Sigma- Aldrich, Y0503, USA) before trypsinization. The cell aggregates were generated in serum-free medium that included DMEM/F12, KOSR, bFGF (Royan institute, 12-280411, Iran), L-glutamine, nonessential amino acids, at seven days post-culture. To prevent apoptosis, 10 μM of ROCK inhibitor was added on the first two days of culture. For passaging, iPSCs and ES cell aggregates were incubated with 10 μM ROCK inhibitor for 2 hours prior to trypsinization then washed with PBS (Gibco, 14287-072, USA) and treated with 0.05% trypsin and 0.53 mM EDTA (Gibco, 25300-054, USA) at 37˚C, for 4-5 minutes. The enzyme was removed and colonies were gently pipetted and re-plated on six-well ultra-low-attachment plates (11).

### Differentiation of iPS and ES cells

**Fig 1:**
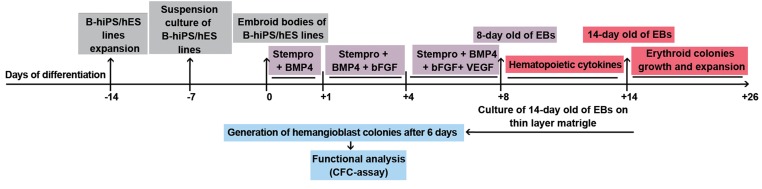
Schematic steps of erythroid differentiation by hiPSCs and hESCs. B-hiPS; Bombay human-induced pluripotent stem, hES; Human embryonic stem, BMP; Bone morphogenetic protein, bFGF; Basic fibroblast growth factor, VEGF; Vascular endothelial growth factor hiPSCs; Human induced pluripotent stem cells, hESCs; Human embryonic stem cells and EBs; Embryoid bodies.

As shown in figure 1, seven-day-old aggregates were cultured in ultra-low attachment six-well plates in the presence of aggregation media that consisted of Stem Pro-34 (Gibco,10639-011,USA) supplemented with 100 IU/ml penicillin, 100 mg/ml streptomycin (Invitrogen, USA), 10 ng/mL bone morphogenetic protein 4 (BMP-4) (R&D Systems, 314-BP, USA), 2 mM glutamine, 4×10^-4^ mM on othioglycerol (MTG) (Sigma-Aldrich, 018K08122, USA), and 50 μg/mL ascorbic acid (Sigma-Aldrich, A4403, USA). The aggregates were incubated at 37˚C in 5% CO_2_. After 24 hours, half of the media was carefully removed and replaced with fresh aggregation media supplemented with 5 ng/mL hbFGF (induction media 1) and cells were incubated for 72 hours. After incubation, cells were harvested and re-suspended in induction media1, which also contained 10 ng/mL vascular endothelial growth factor (VEGF) (R&D Systems, 293-VE, USA) for an additional four days. To generate hemangioblast colonies, 14-day-old aggregates were plated in Iscoveʼs Modified Dulbeccoʼs Medium (IMDM) (Biowest, Lo192-500, France) with 1% methylcellulose (Sigma-Aldrich, 274429, USA) supplemented with 10% FBS, 2 mM L-glutamine, 50 μg/mL ascorbic acid, 4×10^-4^ M MTG, 150 μg/ mL holo-transferrin (Sigma-Aldrich, T0665, USA), 1 ng/mL hbFGF, 10 ng/mL hVEGF, 100 ng/mL human stem cell factor (hSCF) (R&D Systems, 255- SC, USA), 20 ng/mL human interleukin-6 (hIL-6) (R&D Systems, 206-IL, USA), 2 U/mL human Erythropoietin (hEPO) (R&D Systems, 287-TC, USA), 40 ng/mL hIL-3 (R&D Systems, 203-IL, USA), and 25 g/mL human Insulin-like Growth Factors I (hIGF-I, R&D Systems, 291-G1,USA). Plated aggregates were maintained at 37˚C in a 5% CO_2_ incubator for six days. For direct differentiation of erythroid cells, 14-day-old aggregates were plated in IMDM with 1% methylcellulose, 10% FBS, 2 mM L-glutamine, 100 ng/mL hSCF, 2 U/ mL hEPO, 5 ng/mL hIL-6, 40 ng/mL hIL-3, 40 ng/ mL recombinant human thrombopoietin (rhTPO) (R&D Systems, 288-TP, USA), 25 ng/mL hIGF- 1, 10 ng/mL hVEGF, and 1 ng/mL recombinant human granulocyte macrophage colony-stimulating factor (hGM-CSF) (R&D Systems, 215-GM, USA) (12). Plated aggregates were maintained at 37˚C in a 5% CO_2_ incubator for 12 days.

### Phenotypic analysis of ES and iPS cell-derived blast cells

We evaluated the cellsʼ differentiation stages with monoclonal PE-conjugated antibodies against human KDR, CD34, GPA (Glycophorin A), fetal hemoglobin, and FITC-conjugated antibodies against CD31and CD71. All antibodies were obtained from BD Pharmingenand used for immunophenotyping. The results were determined by BD FACS Calibur and analyzed with WinMDI version 2.9 software.

### Clonogenicity potential of B-hiPSC and HSCderived cells

For evaluation of colony formation capability, we plated 14-day-old aggregates onto a thin layer of matrigel in 96-well plates and cultured them in IMDM medium supplemented with 10% FBS, 10% horse serum, 2 mM L-glutamine, 4×10^-4^ M MTG, 150 μg/ml holo-transferrin, 5 ng/ml bFGF, 10 ng/mL hVEGF, 100 ng/mL hSCF, 20 ng/mL hIL-6, 2 U/mL hEPO, and 25 ng/mL hIGF-1 for six days. Subsequently, six-day-old grape-like blast cells were isolated and platedon methylcellulose base media for 14 days up to 28 days at 37˚C in a 5% CO_2_ incubator.

### Immunocytochemistry

Differentiated cells and colonies in methylcellulose were washed with PBS and fixed in 4% paraformaldehyde (PFA) for 15 minutes, permeabilized with 0.2% triton X-100 for 30 minutes for primary antibody, anti CD31 (1:100, BD, 555849) for endothelial cells was performed for 1 hour at 37˚C. Cells were then washed and incubated with FITC-conjugated secondary antibody, anti-mouse IgG (1:100, BD, 04611) as appropriate, for 1 hour at 37˚C, then washed. PE-conjugated human anti fetal hemoglobin (1:500.BD, 560041) was used for staining erythroid cells. Incubation of cells was performed for 30 minutes at 37˚C. Following the development of distinct adherent populations, Low Density Lipoprotein from Human Plasma, Acetylated, DiI complex (DiI-AcLDL, 5 μg/ mL) (Biomedical Technologies, Stoughton, MA) was added to the media for 2 hours. The cultures were then washed in PBS and the cells were fixed with 4% PFA, 3% sucrose in PBS for 20 minutes at room temperature. Nuclei were stained with 4, 6 Diamidino-2-phenylindole (DAPI) (Sigma, D8417, USA). Finally, cells were analyzed under a fluorescent microscope (Olympus, Japan).

### Gene expression analysis

RNA was extracted from the different samples of iPSC and ES cell aggregates, at days 8 and 14 of differentiation using TRIzol Reagent (Invitrogen, USA). Total RNA was treated with DNase I to remove genomic DNA contamination. Two micrograms of total RNA was used for the reverse transcription reaction with the first strand cDNA synthesis kit (fermentas, UK) and random hexamer primer, following manufacture’s instruction. Quantitative polymerase chain reactions (PCR) were set up in three biological replications with the Power SYBR GreenMaster Mix (Applied Biosystems, USA) and analyzed with the 7500 real-time (RT) PCR system (Applied Biosystems, USA). Expression values were normalized to the average expression of the housekeeping gene Glyceraldehyde 3-phosphate dehydrogenase (GAPDH). The primer sequences are presented in Table 1.

**Table 1:** Real-time polymerase chain reaction (RT-PCR) primers


Gene	Primer sequence	Annealingtempera-ture (˚C)

**GAPDH**	F: 5´-CTCATTTCCTGGTATGACAACGA-3´	60
	R: 5´-CTTCCTCTTGTGTTGCT-3´	
**RUNX1**	F: 5´-TCGGCTGAGCTGAGAAATG-3´	60
	R: 5´-GATGTCTTCGAGGTTCTCGG-3´	
**TAL1**	F: 5´-GAGGTAATTCCCAGCCATTGAC-3´	60
	R: 5´-GAAGCCGAGGAAGAGGATGC-3´	
**c-KIT**	F: 5´-ATTGTTCTGTGGACCAGGAG-3´	60
	R: 5´-GGTTGTTGTGACATTTGCTG-3´	
**CD34**	F: 5´-CAACAACGGTACTGCTACCC-3´	60
	R: 5´-AAACATTTCCAGGTGACAGG-3´	
**Hbα**	F: 5´-ACGGCTCTGCCCAGGTTAAG-3´	60
	R: 5´-TTGAAGTTGACCGGGTCCAC-3´	
**Hbβ**	F: 5´-TCTGTCCACTCCTGATGCTG-3´	60
	R: 5´-GATGCTCAAGGCCCTTCATA-3´	
**Hbγ**	F: 5´-ACTATCACAAGCCTGTGGGG-3´	60
	R: 5´-GAATTCTTTGCCGAAATGGA-3´	


GAPDH; Glyceraldehyde 3-phosphate dehydrogenase, RUNX1; Runt-related transcription factor 1, TAL1; T cell acute lymphoblastic leukemia, c-KIT; Stem cell growth fac-tor receptor (tyrosine-protein kinase Kit), CD34; Clusters of differentiation 34 and Hb; Hemoglobin.

### Statistical analysis

Data are presented as mean ± standard deviation. Multiple comparisons were performed with the repeated measure test. Differences were considered statistically significant at p≤0.01 or p≤0.05.

## Results

### Differentiation of B-hiPSCs and hESCs into hemangioblast progenitors

**Fig 2:**
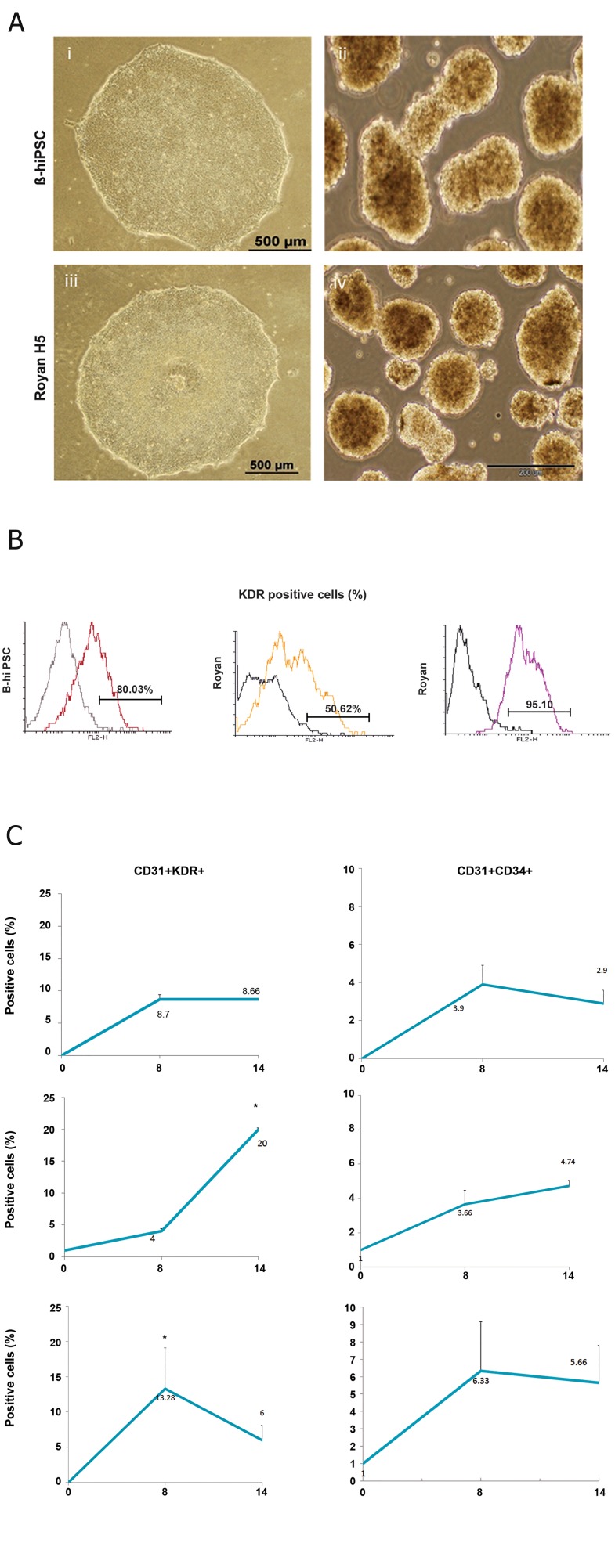
Morphology, phenotype and mRNA analysis of B-hiPSC-derived cells before and after differentiation. A. Photograph of B-hiPSC and ESC colonies on a thin layer of matrigel (i, iii). Aggregate formation from B-hiPSC and ESC colonies under suspension conditions (ii, iv). B. Flow cytometry analysis was used to investigate KDR expression differences between B-hiPSC and ESC lines on day 14 of differentiation. C. Flow cytometry analysis showing the expression of early hematopoietic surface antigens in aggregates at days 8 and 14 of differentiation in iPSC and ES cell lines. Undifferentiated iPSC and ES cells (day 0) were used as a negative control (n=3). B-hiPSC; Bombay human-induced pluripotent stem cells, ESC; Embryonic stem cell, CD; Clusters of differentiation, KDR; Kinase insert domain receptor and *; P≤0.05.

The initial step in erythroid differentiation is hemangioblast formation, for which we expanded B-hiPS and hES cells on matrigel-coated plates. Cells in both lines formed compact colonies (Fig 2Ai, iii). Then, the suspension culture was used for cell proliferation and aggregate formation (Fig 2Aii, iv). We used a two-step method for hemangioblast differentiation. The first step lasted for eight days and the combination of BMP-4, b-FGF, and VEGF was added between days 1 and 8 in serum-free medium. In the second step, the culture was allowed to continue until day 14 in the presence of VEGF, SCF, IL-6, EPO, IL-3, and IGF-1. Co-expression of kinase insert domain receptor (KDR or FLK-1), clusters of differentiation 34 (CD34) and CD31 has been shown to determine early stage hematopoietic development (13-15), therefore we tested the cells for expressions of those markers. According to our results, undifferentiated ES cells and iPSCs expressed KDR at low levels, while culture condition changed to erythrocyte differentiation media, expression of KDR significantly increased on day 14 in all lines (Fig 2B, p≤0.01). However this expression was prominent in differentiated iPSCs (80%) and RH5SCs (95%), when compared with RH- 6SCs (50%).

As shown in figure 2B, co-expression of KDR and CD3l was not detected in undifferentiated cells. During eight days of culture, the percent of CD31^+^KDR^+^ (8.7%) and CD31^+^CD34^+^ (3.9%) cells significantly increased in the iPSC cell lines in addition to the significant increase that was also noted for CD31^+^KDR^+^ (13.28%) and CD31^+^CD34^+^ (6.33%) cells in the RH5 ES cell lines (p≤0.05). Expression of these cells persisted through 14 days of culture in iPSC lines and RH5 ES cells. In contrast, CD31^+^KDR^+^ and CD31^+^CD34^+^ cells increased after 14 days of culture in RH6 ES cells (Fig 2B). However, the CD31^+^KDR^+^ cells showed a more prominent increase than CD31^+^CD34^+^ cells. We observed that all CD31^+^KDR^+^ cells expressed CD34^+^ surface markers which might have been related to their hemangioblast origin (data not shown). A comparison of all cell lines for expression of hemangioblast markers showed that the pattern of expression was similar in both the ES cell and iPSC lines, with the exception of RH6 that had a significant increase in expression of CD31^+^ KDR^+^ up to day 14 (p≤0.05).

**Fig 3:**
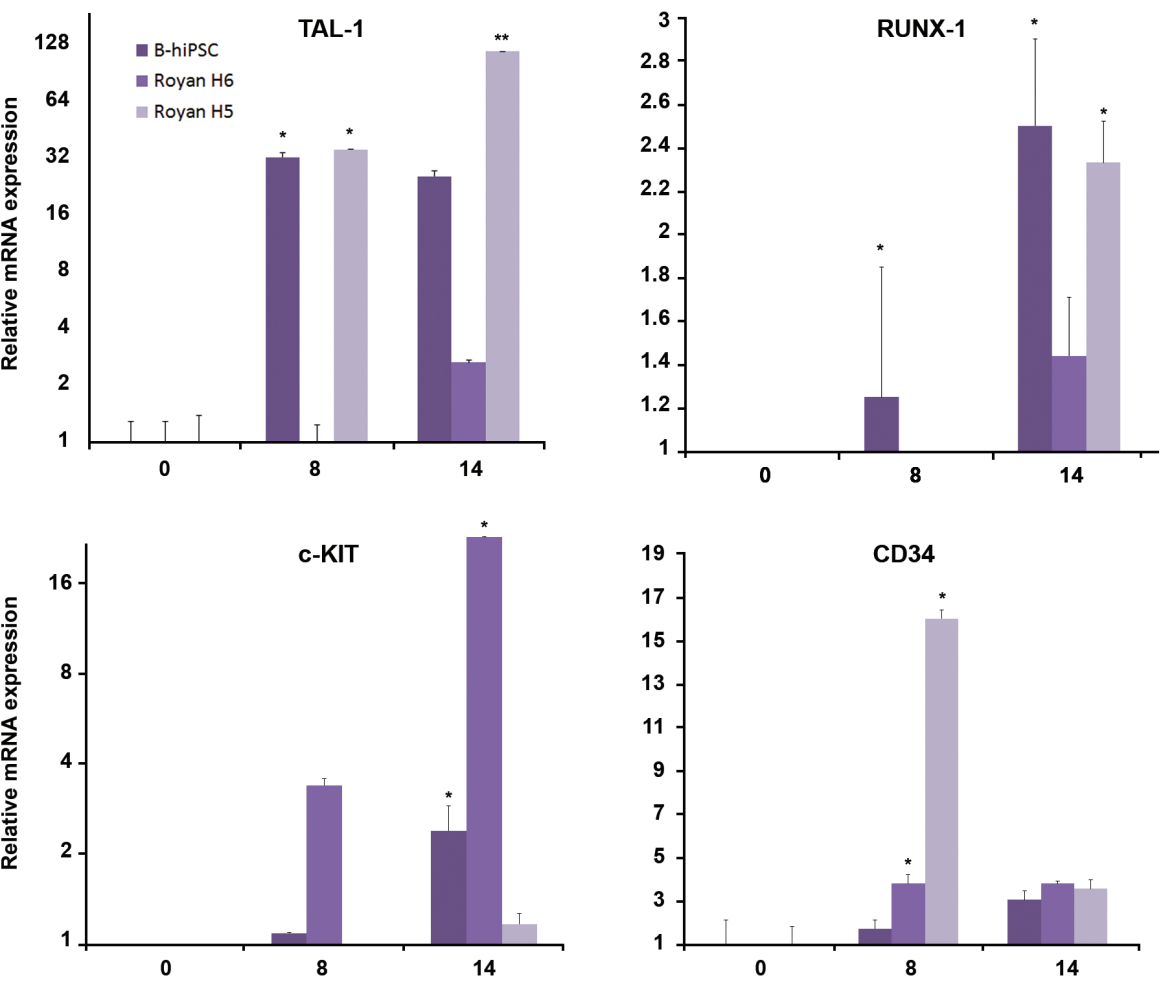
Quantitative RT-PCR. Total mRNA of cells from indicated day were extracted and analyzed for expression of specific genes (Table 1) by quantitative RT-PCR using the 2-ΔΔCT method (n=3). B-hiPSCs; Bombay human-induced pluripotent stem cells, CD; Clusters of differentiation, RT-PCR; Real-time polymerase chain reaction, **; P≤0.01 and *; P≤0.05.

To confirm the above data, the expressions of major hematopoietic genes, *CD34, RUNX-1, c-KIT*, and *SCL (TAL-1)* (16-20) were assessed in ES cells and iPSCs on days 8 and 14 of differentiation by quantitative RT-PCR. Our results determined that the expression of *TAL-1, RUNX-1, c-KIT*, and *CD34* up-regulated at day eight and continued or increased up to day14 in both ES cells as well as iPSCs. Expression of CD34 decreased only in RH5 significantly until day 14 (Fig 3). Therefore, we proposed that the ES and iPS cells in the twostep protocol differentiated into hemangioblasts.

### Identification of hemangioblast functionality

**Fig 4:**
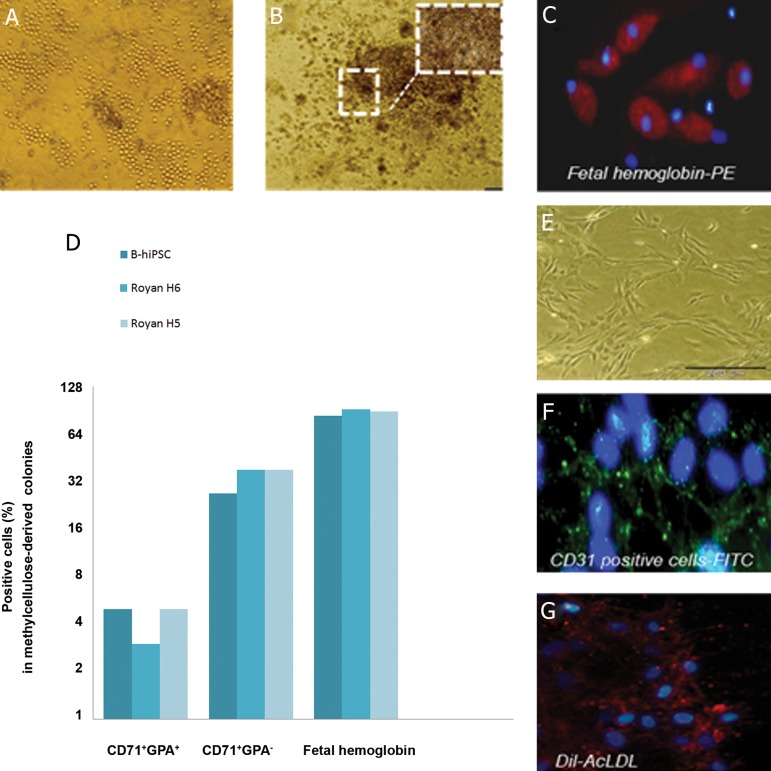
Clonogenicity of blast colonies. A. Photograph of grape-like blast colonies generated from day14 aggregates on a thin layer of matrigel. B. Photograph showing erythroid and non-erythroid colonies (mixed-colonies) which differentiated from six-day-old blast colony in methylcellulose base media in B-hiPSC line. C. Immunostaining of erythroid cells by anti-human fetal hemoglobin confirmed the expression of fetal hemoglobin that had been shown by flow cytometry. Nuclei were stained with DAPI (magnification: ×40). D. Mixed colonies picked from CFU-culture. Expression of erythroid-specific marker CD71 (transferring receptor), CD235 (GPA) and fetal hemoglobin as analyzed by flow cytometry. E-G.Endothelial cells appeared from B-hiPSCs; expression of CD31 markers shown by green fluorescence and LDL uptake by red fluorescence. Nuclei were stained with DAPI (magnification: ×40). B-hiPSCs; Bombay human-induced pluripotent stem cells, CD; Clusters of differentiation, GPA; Glycophorin A and DAPI; 4,6 Diamidino-2-phenylindole.

As previously mentioned, cells from earlystage aggregates (14-day) were cultured in conditions known to support the growth of blast colonies. As shown in figure 4A, colonies with grape-like morphology of hemangioblast colonies were detected in ES and iPSC lines after seeding on a thin layer of matrigel for six days. Cells isolated from these colonies at days 3, 4, 5, and 6 were sub-cultured on methylcellulose to form hematopoietic progenitor cells. As shown in figure 4B, six-day-old colonies formed two types of cells on methylcellulose, adhesive and non-adhesive (or loosely adhesive). Interestingly, non-adhesive cells formed small colored colonies, their color changed to red pale and more than 80% of them expressed fetal hemoglobin (Fig 4B, C). It seems the culture includes mixed cells. For further evaluation of the erythroid cells, we chose colonies cultured on methylcellulose to be pooled and analyzed for CD71 and GPA expressions by flow cytometry. According to our findings, about 5-8% of cells from all lines expressed CD71^+^ GPA^+^ (p≤0.05). There was a similar pattern of CD71^+^ GPA^+^ and fetal hemoglobin expression seen in iPSCs and RH5SCs. However, there was a difference in expression of CD71^+^GPA^-^ in the ES cell group (38%) compared to the iPSC group (27%) (Fig 4D). As during erythroid development, the expression of CD71 happens earlier, followed by co-expression with GPA. In mature erythrocytes, expression of GPA increased (21), therefore we have proposed that to promote erythrocyte maturation *in vitro*, conditions should be conducive to support more erythroid maturation.

We sought to determine the characteristics of the adhesive cells which developed from sixday- old hemangioblast-like cells. There were endothelial-like cells in our culture (Fig 4E), therefore we evaluated CD31 expression and uptake of Dil-AcLDL, both of which are specific for endothelial cells. Immunostaining of the blast colony-derived adhesive population revealed that over 30% were positive for CD31 surface antigen (Fig 4F) and had the potential for uptake of Dil-AcLDL (Fig 4G) in all lines. However, the condition of culture was supported hematopoietic coloniesfurther, hence, the adhesive cells could not proliferate more. These results have demonstrated the capability of hiPS and ES cells to differentiate into hemangioblasts, erythroid, and endothelial lineages under our differentiation system.

### Direct differentiation of 14-day aggregates into fetal-like erythroid cells

**Fig 5:**
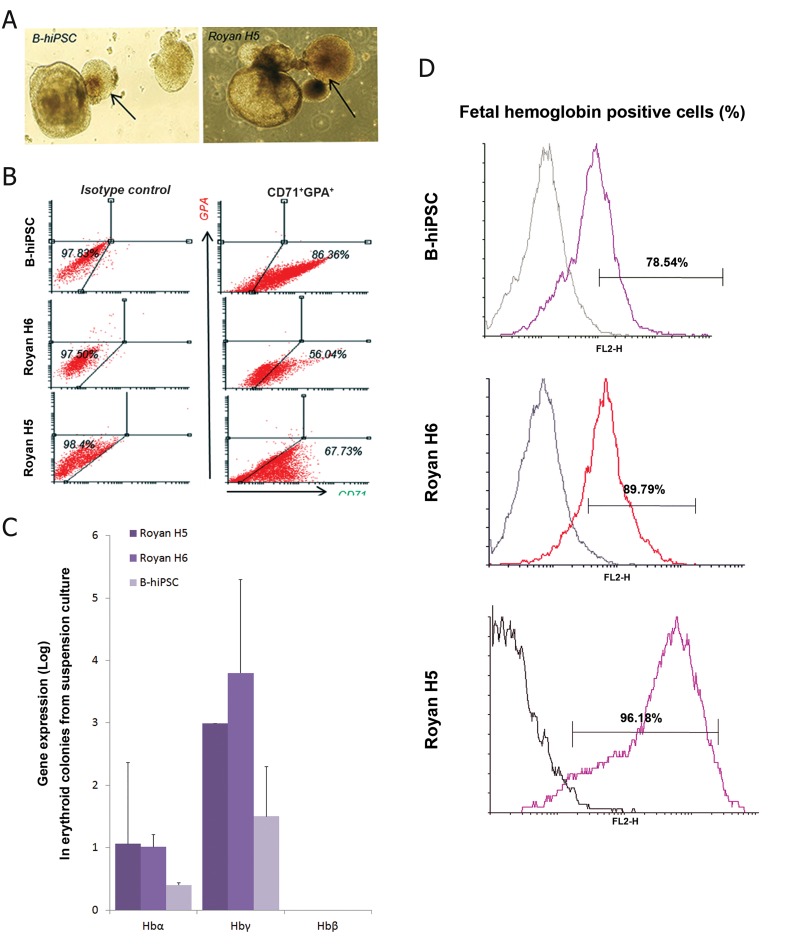
Surface markers expression and mRNA analysis of erythroid cells generated from direct differentiation of 14-day aggregates. A. Photographs of erythroid colonies in iPSC and ES cell lines that indicated a change in color during suspension culture, as indicated by arrows (magnification: ×20). B. Flow cytometry analyses showing expression patterns of GPA and CD71 in both lines, (n=3).*; P≤0.05. C. The mRNA levels of α, γ and β-hemoglobin in erythroid cells in iPSC and ES cell lines. D. Flow cytometry analysis showing fetal hemoglobin expression in iPSC and ES cell lines. B-hiPSCs; Bombay humaninduced pluripotent stem cells, CD; Clusters of differentiation, GPA; Glycophorin A, Hb; Hemoglobin and ES; Embryonic stem.

Our previous results determined that 14-day aggregates expressed KDR, CD31 and CD34; therefore, these aggregates were chosen for direct erythroid differentiation without passage through the hemangioblast formation step. Aggregates cultured in the suspension media that included SCF, IL-6, VEGF, IL-3, EPO, GMCSF, and IGF-1. At 12 days post-culture, colors of colonies changed which indicated with increased expression of hemoglobin in some parts of the colonies (Fig 5A). For obtaining more accurate results, we continued the culture of these aggregates up to day 16, then characterized their erythroid surface antigens, intracellular proteins, and globin genes. According to phenotype analysis, co-expression of CD71/ GPA, which is related to erythroid maturation (16) was not detected. In contrast, we observed that both ES cells expressed approximately 40% CD71^+^GPA^-^ (Fig 5B) compared to more than 80% of erythroid cells that were derived from iPSCs which expressed CD71^+^GPA. Up-regulation of α- and γ-globin mRNA was detected in both ES cells related to iPSCs. However, expression of β-globin was not detected in both lines (Fig 5C). Also, there was approximately an 80% expression of fetal hemoglobin in RH6 line and iPSCs compared with 60% observed in the RH5 line (Fig 5D). These results confirmed that the fetal like erythroid cells produced from both lines in this experiment and also suggested these conditions are more supportive for fetal not adult characteristic. It appears that the combination of the aforementioned cytokines induced early erythroid differentiation, mainly EPO, which played an essential role in the emergence of erythroid cells. The synchronous presence of two specific cytokines, SCF and EPO, produced a signal that markedly affected over-expression of α- and γ-globin genes, particularly in both ES cells.

## Discussion

Although transfusion of RBCs is a well-established cellular therapy; the lack of healthy donors, possibility of human viral infection and increasing the requirement for immunological matching limit its use. However, new discoveries in stem cell research and introduction of pluripotent stem cells (PSCs), such as ES cells (22, 23) and iPS cells (8, 24, 25) with their potential to form any cell type *in vitro*, have been sought as possible sources to candidate for the production of unlimited numbers of erythroid cells. Most studies have shown hES cells and hiPS cells cultured in the presence of animal serum and OP9 or MS-5 mouse fibroblasts as feeder layers (26-30) in order to produce erythrocytes. Few data exists for hiPSC differentiation into erythrocytes in feeder-free and serumfree medium.

Thus, this study employed the use of feederfree culture for erythrocyte differentiation *in vitro* with the intent to propose new, unlimited cell sources that can be an appropriate source for those who need cell therapy in future. For first time, we used iPSCs which have been derived from adult cells that carry the Bombay phenotype which fails to express ABH antigens on RBCs (31, 32). These cells have been used to generate histocompatible erythroid cells and introduce a universal red blood source that is not patient-specific and compatible with all patients’ immune systems. We have attempted to examine the potential for erythroid differentiation of B-hiPSCs derived from adult cells that carry the Bombay phenotype, and then we compared their capability with ES cells.

Previous research in our lab has shown that ES cells and iPSCs could be maintained and expanded as aggregate suspensions over an extended period and then induced for specific differentiation into cardiac and hepatic cells (11). In this study, we used a feeder-free suspension culture and have produced aggregates that underwent induction of differentiation toward erythroid cells in the presence of several cytokines which are necessary for erythroid differentiation in a suspension culture.

Our results determined that B-hiPS, hRH5SC and hRH6SC have expressed the crucial genes *TAL-1, RUNX-1, c-KIT* and *CD34* which are essential during early development of hemangioblasts in humans (16, 18, 33, 34) and can differentiate to hemangioblastsat the beginning of differentiation which is concomitant with upregulation of *TAL-1, RUNX-1* and *c-KIT* genes that correlated with their mesodermal-hematopoietic properties.

According to our analyses, KDR was expressed on undifferentiated iPSCs and ES cells, and then it increased between days 8 and 14 of differentiation. KDR as a tyrosine kinasereceptor binds to its ligand, VEGF and KDR/ VEGF activates expression of genes which are crucial in erythroid development. In primitive streak–stage embryos, KDR expression is first detectable in cells within and exiting the primitive streak as well as in the extra-embryonic mesoderm that is crucial for development of cardiac, endothelial and hematopoietic progenitor cells (35, 36). It seems that the KDR^+^ population involves hematopoietic along with cardiac and endothelial progenitors. Additionally the combination of BMP4 and VEGF increased the numbers of KDR-positive cells in 14-day embryoid bodies (EBs) (37). Our current study also showed that iPSC- and ES cell-derived hemangioblasts expressed KDR. Thus expression of KDR increased progressively up to day 8 of differentiation, rather than expressions of CD31 and CD34. In contrast to the other lines, co-expression of CD31KDR and CD31CD34 markers increased significantly in RH6 line on day 8 then persisted up to day 14, although co-expression of these surface antigens did not changed significantly at day 14. A number of previous studies have shown the development of hES cell-derived cell types with hemangioblast properties. Some research has shown that the CD31^+^VE-cadherin^+^KDR^+^CD45^-^ population in day-ten aggregates displayed the potential to generate both hematopoietic and endothelial progeny (38). Although we did not examine the expression of CD45, however there was expression of hemangioblast-specific markers. Subsequently, we demonstrated that iPSCs were similar to ES cell lines by their ability to differentiate into erythroid cells, the type of globin expression, surface antigen expression, and the ability to form mixed colonies. However the type of ES cells can also af fect the results.

A new finding in our results was the formation of endothelial-like cells at the time hemangioblasts were formed. The earliest stage of hematopoietic development in the human and mouse embryo begins in the yolk sac, within blood islands that consist of emerging primitive erythroblasts surrounded by endothelial cells. Consequently, researchers hypothesize that these lineages share a common origin, a progenitor known as hemangioblasts (39-41). Interestingly, when mouse-derived hemangioblasts are cultured in methylcellulose media, these progenitors generate immature blast colonies that display both hematopoietic and vascular potentials (42). The cell that produces these colonies, the blast colony-forming cell (BLCFC) or hemangioblast, expresses the receptor tyrosine kinase *Flk-1* and the mesodermal gene T (brachyury), which demonstrates that it represents a population undergoing mesoderm specification to hematopoietic and vascular lineages (13, 43, 44).

Thus we have suggested that our culture method induced mesodermal-hematopoietic progenitors (36) which easily gave rise to endothelial and hematopoietic progeny, which is similar to the mixed colonies obtained from CD133 or CD34 and mononuclear cells present in bone marrow, peripheral or cord blood progenitors.

Although co-expression of CD71/GPA was shown in erythroid cells, expression of fetal hemoglobin was significantly high. Interestingly, most cells in the mixed colonies were erythroid and had high expression of hemoglobin. The duration of the culture was greater than previous and the number of cells that expressed hemoglobin increased (data not shown). It seemed that the long-term presence of VEGF was effective in increasing the erythroid population (45). We did not examine the expression of megakaryocytic lineage markers in mixed colonies, although studies have indicated that erythroid and megakaryocytic lineage commitment take place together and potentially arise from a common precursor population (38). Given that adherent cells express CD31 surface antigen, possibly they arose from bipotential cells as nominated hemangioblast that differentiated from iPS and ES cells in our experiment. However, they need to be cultured as single-cells for more detailed characterization.

We have attempted to differentiate iPSCs and hES cells directly into erythroid cells in suspension culture over a short period of time. Our results revealed that iPS and hES cells could produce erythroid cells in this system. Although the majority of cells were hemoglobinized, there was a low-level co-expression of CD71/ GPA. Possibly the presence of FBS promoted the generation of CD71^+^GPA^-^ cells and reduced the number of cells that expressed GPA. According to Chang et al. (3), when non-adherent cells were expanded in serum-free medium in the absence of FBS they gave rise to a higher frequency of GPA cells.

According to our results, erythroid cells expressed high levels of fetal hemoglobin. mRNA expression analyses also confirmed that they expressed α- and γ-globin, where remarkably, the expression level of γ-globin was more. This was possibly related to the presence of several cytokines as EPO, SCF. It has been shown that the combination of EPO, SCF and transforming growth factor-beta (TGF-β) signal transduction produce a marked increase in γ-globin transcript and protein expression (46). Erythroblasts cultured in the presence of these cytokines reveal a significant enhancement of fetal hemoglobin (HbF) without significant effect on erythroblast maturation (47, 48). SCF also has anti-apoptotic effects on cultured erythroblasts (49, 50). Due to the lack of mature erythroid cells, we have proposed that the cytokine signals used in our test most likely have changed the expression levels of transcription factors essential for erythroid commitment and have not affected terminal differentiation or maturation of erythroblasts. Accordingly, our culture conditions have supported increase in fetal hemoglobin expression.

## Conclusion

**Fig 6:**
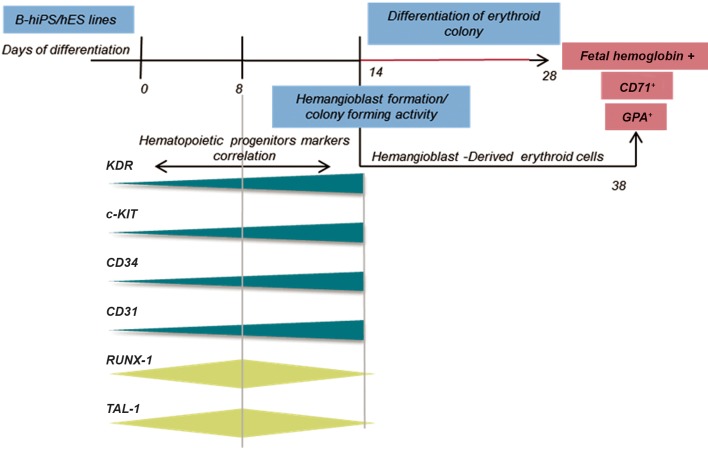
Summary of cellular and molecular events during differentiation of iPSCs and hESCs. Developmental progression of hemangioblast and erythroid gene expression during differentiation and correlation of key genes and markers. B-hiPS; Bombay human-induced pluripotent stem, hES; Human embryonic stem, CD; Clusters of differentiation and GPA; Glycophorin A.

Finally, our study provided evidence that B-hiPS differentiated to erythroid cells similar to ES cells and produced a population with KDR^+^CD31^+^CD34^+^ characteristics. In addition, they were able to produce colonies with hemangioblast properties (Fig 6) and in the final step, they differentiated to fetallike erythroid cells.

Our ability to produce erythroid cells with a fetal phenotype from iPSCs and ES cells might assist with studies on the development of early erythropoiesis in humans and be of practical use for examining therapies for different blood disorders, particularly hemoglobinopathies characterized by insufficient production of β-globin chains due to mutations that affect the β-globin gene complex. However, our inability to produce adult RBCs from iPSCs or ES cells will affect possibility of these experiments that exposure beneficial in the near future. It seems that production of RBCs with an adult phenotype from pluripotent cells is a critical step and an issue that remains unresolved, thus necessitating the need to develop and use more advanced techniques.
